# The Catheter in Abdomino-Pelvic Operations in Women

**Published:** 1903-01-10

**Authors:** 


### THE CATHETER IN ABDOMINO-PELVIC OPERATIONS IN WOMEN.

Mr. Stanmore Bishop[1] says that there appears to be a general impression amongst medical men, and still more among nurses, that no case is properly prepared for abdominal or vaginal section unless the catheter has been passed just before removal to the operating theatre. From this view he dissents. It is of course easy to show that in certain cases of impacted fibroid, or fibroid of the broad ligament it might not be possible to empty the bladder sufficiently in the natural manner, and a catheter might then be required, but such questions are exceptional and do not touch the point raised, namely, that in the routine pre-operative use of the catheter a danger, that of septic cystitis, which is very real is incurred for no apparently sufficient reason. Again, in regard to post-operative catheterism, when the operation has been done for some condition of the bladder itself catheterism may be not only advisable but imperative. As to this in each case the surgeon must decide. But in regard to the routine use of the catheter whenever difficulty occurs in micturition after operation, Mr. Stanmore Bishop urges the importance of avoiding the production of cystitis after operation in women, since it adds greatly to the risk, and may utterly spoil the perfect result otherwise obtainable, and adds that the great cause of such cystitis is catheterisation. Routine pre-operative catheterisation is unnecessary and futile, while post-operative catheterisation can, in the majority of instances, be avoided. The bladder will empty of itself if time be allowed, and when once this has occurred no further question of the catheter being required is likely to arise.

[1] Practitioner, December.

